# Kimura Disease in an Adolescent With Type 1 Diabetes Mellitus: A Rare Co-occurrence of T Helper 2-Driven Eosinophilic Lymphadenopathy and Autoimmune Endocrinopathy

**DOI:** 10.7759/cureus.116339

**Published:** 2026-09-16

**Authors:** Khalid Al Khathlan, Abdulrahman Aldoukhi, Abdulrahman M Aldhilan, Abdulaziz Alroqi, Ahmed Almukhlifi, Ziad G Alanazi, Saeed AlShieban

**Affiliations:** 1 Division of Rheumatology, Department of Medicine, Ministry of National Guard Health Affairs, Riyadh, SAU; 2 Department of Rheumatology, King Abdulaziz Medical City, Riyadh, SAU; 3 Department of Hematology, King Abdulaziz Medical City, Riyadh, SAU; 4 Department of Internal Medicine, Ministry of National Guard Health Affairs, Riyadh, SAU; 5 College of Medicine, King Saud Bin Abdulaziz University for Health Sciences, Riyadh, SAU; 6 Department of Pathology, King Abdulaziz Medical City, Ministry of National Guard Health Affairs, Riyadh, SAU; 7 Department of Pathology, King Abdullah International Medical Research Center, Riyadh, SAU

**Keywords:** adolescent, autoimmunity, eosinophilia, eosinophilic lymphadenopathy, excisional biopsy, histopathology, immunoglobulin e, kimura disease, lymphadenopathy, type 1 diabetes mellitus

## Abstract

Kimura disease (KD) is a rare, chronic T helper 2 (Th2)-driven inflammatory disorder characterized by painless head-and-neck lymphadenopathy or subcutaneous masses, peripheral and tissue eosinophilia, and markedly elevated serum immunoglobulin E (IgE). It predominantly affects young men of Asian descent. Although KD is regarded as an allergic or immune-dysregulatory rather than a classically autoimmune condition, it has been described in association with nephrotic syndrome, suggesting an underlying systemic immunologic disturbance. Its co-occurrence with type 1 diabetes mellitus (T1DM), an archetypal organ-specific autoimmune disease, has not, to our knowledge, been previously reported.

A 17-year-old male with T1DM diagnosed at nine years of age presented with a painless, slowly enlarging right submandibular mass of 18-24 months' duration, without fever, night sweats, or weight loss. Examination revealed a firm, non-tender 3 × 3 cm right submandibular swelling. Investigations showed leukocytosis with marked eosinophilia and serum IgE persistently above 5000 IU/mL. Neck computed tomography demonstrated a 4.2 × 2.3 cm inhomogeneously enhancing submandibular mass with ipsilateral cervical lymphadenopathy, considered suspicious for lymphoproliferative disease. An extensive infectious work-up was negative. Additionally, serology revealed a broadly negative autoantibody profile. There was significant IgE-mediated food allergy and chronic rhinosinusitis, on a background of a strong family history of autoimmune disease. Two sequential ultrasound-guided core-needle biopsies were non-diagnostic, showing only reactive lymphoid hyperplasia with focal microabscess and granuloma formation. Excisional lymph node biopsy demonstrated preserved follicular architecture with paracortical eosinophilic infiltration, eosinophilic microabscesses, Charcot-Leyden crystals, and vascular proliferation, without malignancy and with negative Epstein-Barr virus (EBV)-encoded small RNA (EBER) and human herpesvirus 8 (HHV-8) staining, diagnostic of KD.

This report describes a classic presentation of KD occurring in an adolescent with established T1DM and a broader personal and familial autoimmune and atopic diathesis. It supports the view that KD may arise within a wider spectrum of immune dysregulation rather than in isolation, and reinforces that excisional biopsy, not needle sampling, is required for timely diagnosis.

## Introduction

Kimura disease (KD) is a rare, chronic inflammatory disorder first described by Kimm and Szeto in 1937 and subsequently named after Kimura T, who characterized it in 1948 as “eosinophilic hyperplastic granuloma” [1]. It predominantly affects young to middle-aged men of East and Southeast Asian descent, though sporadic cases occur worldwide, and typically presents with painless subcutaneous masses or lymphadenopathy of the head and neck, peripheral blood eosinophilia, and markedly elevated serum immunoglobulin E (IgE) [1-3].

The pathogenesis of KD is thought to reflect a dysregulated T helper 2 (Th2) immune response, with local and systemic overproduction of interleukin (IL)-4, IL-5, and IL-13 driving eosinophil recruitment, IgE class-switching, and lymphofollicular hyperplasia. For this reason, KD has traditionally been regarded as an allergic or hypersensitivity-type disorder rather than a classical autoimmune disease. Nevertheless, it has been proposed to represent a manifestation of broader systemic immunologic disturbance, and has been reported in association with nephrotic syndrome, its most frequent systemic complication [1,4,5].

Type 1 diabetes mellitus (T1DM), by contrast, is an archetypal organ-specific autoimmune disease driven predominantly by T helper 1 (Th1)-polarized, cell-mediated destruction of pancreatic beta cells. T1DM is well recognized to cluster with other autoimmune conditions, most commonly autoimmune thyroid disease and celiac disease, reflecting shared genetic susceptibility and a general predisposition to loss of immune tolerance [6]. The historical Th1/Th2 dichotomy has, however, been substantially revised, and Th1- and Th2-mediated disorders are increasingly recognized to coexist within the same individual [7].

We report an adolescent with longstanding, established T1DM who developed a clinically and histologically classic presentation of KD. To our knowledge, this co-occurrence has not previously been described. We highlight the diagnostic pathway, including the failure of successive core-needle biopsies, and discuss the case in the context of the patient's broader personal and familial autoimmune and atopic background.

## Case presentation

Patient information

A 17-year-old male, a known case of T1DM diagnosed at approximately nine years of age, presented to the primary healthcare with an insidiously enlarging, painless right submandibular swelling of approximately 18-24 months' duration. His diabetes had initially presented with diabetic ketoacidosis and was complicated by recurrent ketoacidotic episodes (three to four per year) until the introduction of insulin-pump therapy, after which glycemic control improved substantially. He was performing well academically and was otherwise developmentally normal.

He denied fever, night sweats, weight loss, recent upper respiratory tract infection, or dental pain, and reported a good appetite. Oral hygiene was fair, without dental caries. There was no history of animal contact, and no compressive symptoms, stridor, hoarseness, or choking.

The family history was notable for a substantial autoimmune burden: a younger sister with T1DM, two maternal cousins with T1DM, a maternal cousin with multiple sclerosis, and a maternal uncle with thyroid disease. There was no family history of celiac disease, vitiligo, malignancy, or hematologic disorder. The parents were non-consanguineous but of the same tribe. The patient was a lifelong non-smoker raised in a non-smoking household, with no pets or mold exposure.

Clinical findings

On examination, there was a solitary, firm, non-tender 3 × 3 cm swelling in the right submandibular region, not mobile, with no overlying skin change. The remainder of the neck examination was unremarkable, with no other palpable lymphadenopathy, and systemic examination was normal. Subsequent head-and-neck clinic assessment described a mobile, fibrotic right level IB lymph node measuring approximately 3 × 4 cm with normal overlying skin; flexible laryngoscopy showed a clear oropharynx, hypopharynx, supraglottis, and glottis, with mild deviation of the uvula to the right (Figure 1).

**Figure 1:**
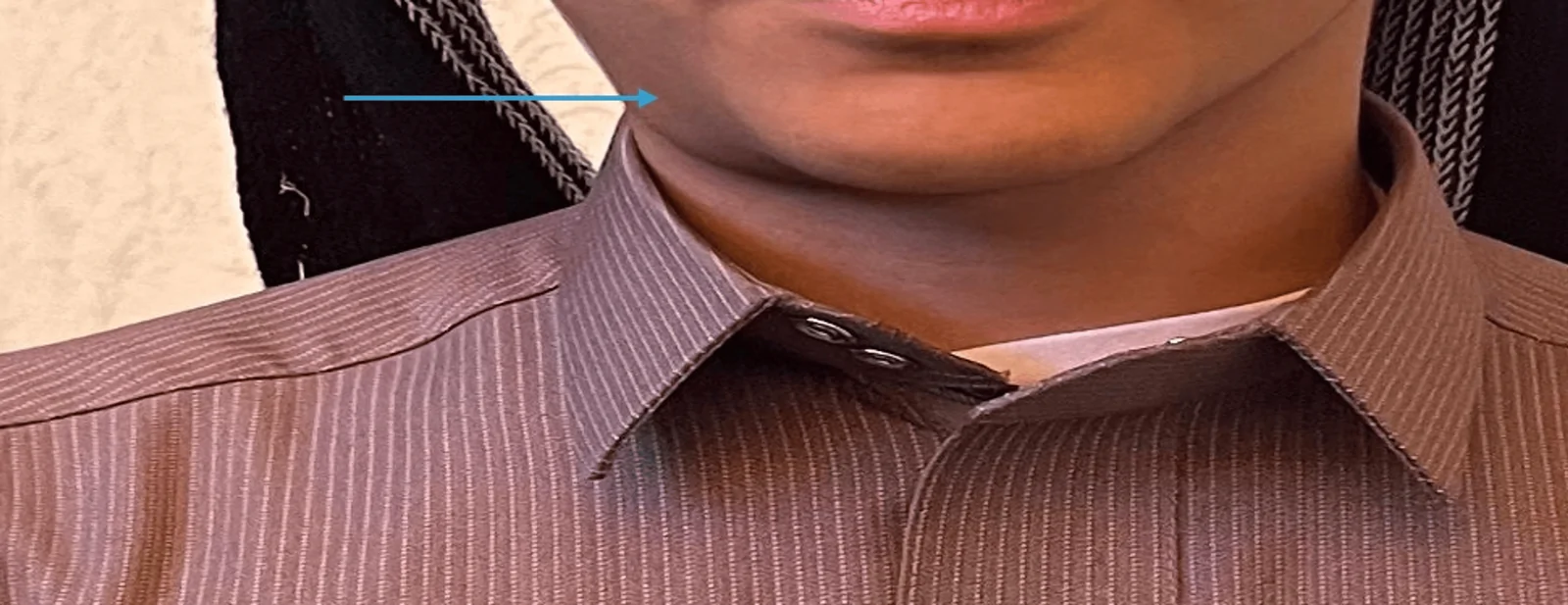
Clinical photograph of the right submandibular region at presentation. The blue arrow indicates the site of the enlarged right submandibular lymph node.

Diagnostic assessment

Complete blood count at presentation demonstrated leukocytosis (13.2 × 10⁹/L) with eosinophilic predominance; absolute eosinophil counts subsequently rose to 8.6 × 10⁹/L, representing up to 30-48% of the differential. Peripheral blood smear showed eosinophilia with adequate neutrophils and mature lymphocytes, with no circulating blasts or abnormal cells identified. Serum total IgE was persistently greater than 5000 IU/mL. Renal and hepatic profiles were normal, and inflammatory markers were unremarkable.

Given the differential of infection and malignancy, an extensive infectious work-up was performed and was negative or non-reactive throughout, including Epstein-Barr virus (quantitative and qualitative), cytomegalovirus, mumps, rubella, *Brucella abortus* and *Brucella melitensis*, human immunodeficiency virus antigen/antibody, Schistosoma antibody, hepatitis B and C serologies, stool examination for ova and parasites, and tuberculosis PCR, acid-fast bacilli staining, and QuantiFERON-TB. Hematologic evaluation, including bone marrow biopsy, confirmed that eosinophils were markedly increased in peripheral blood and bone marrow with unremarkable morphology, and ruled out primary hematologic malignancies such as leukemia or lymphoma.

Serologic testing revealed a broadly negative autoantibody profile, including negative antinuclear antibody, anti-double-stranded DNA by enzyme-linked immunosorbent assay (ELISA), myeloperoxidase-ANCA (antineutrophil cytoplasmic antibody), proteinase-3-ANCA, anti-Sm, anti-RNP, anti-SS-A, anti-SS-B, rheumatoid factor, and anti-cyclic citrullinated peptide antibodies. Complement C3 and C4 were normal, and cryoglobulins were absent. Thyroid function was normal. Aside from persistently elevated IgE, other serum immunoglobulins were within range. The full laboratory panel is summarized in Table 1.

**Table 1:** Summary of laboratory investigations at presentation and follow-up. IU/mL: international units per milliliter; µmol/L: micromoles per liter; U/L: units per liter; mg/L: milligrams per liter; mm/h: millimeters per hour; g/L: grams per liter; mIU/L: milli-international units per liter.

Laboratory parameter	Patient result	Reference range
White blood cell (WBC) count	13.2 × 10⁹/L	4.5–11.0 × 10⁹/L
Absolute eosinophil count (AEC)	8.6 × 10⁹/L	0.0–0.5 × 10⁹/L
Serum total immunoglobulin E (IgE)	>5000 IU/mL	<100–200 IU/mL
Serum creatinine	50 µmol/L	44–88 µmol/L
Alanine aminotransferase (ALT)	10 U/L	<45 U/L
Aspartate aminotransferase (AST)	15 U/L	<34 U/L
Total bilirubin	6.6 µmol/L	<20.5 µmol/L
C-reactive protein (CRP)	1.80 mg/L	<5 mg/L
Erythrocyte sedimentation rate (ESR)	13 mm/h	0–15 mm/h
Antinuclear antibody (ANA)	5.39 units	<20 units
Anti-double-stranded DNA (anti-dsDNA) antibody	80.75 IU/mL	<200 IU/mL
Myeloperoxidase antineutrophil cytoplasmic antibody (MPO-ANCA)	6.0 units	<20 units
Proteinase-3 antineutrophil cytoplasmic antibody (PR3-ANCA)	1.36 units	<20 units
Anti-Smith (anti-Sm) antibody	4.60 units	<20 units
Anti-ribonucleoprotein (anti-RNP) antibody	3.71 units	<20 units
Anti-Sjögren's-syndrome-related antigen A (anti-SS-A) antibody	2.68 units	<20 units
Anti-Sjögren's-syndrome-related antigen B (anti-SS-B) antibody	2.92 units	<20 units
Rheumatoid factor (RF)	<20 IU/mL	<30 IU/mL
Anti-cyclic citrullinated peptide (anti-CCP) antibody	11.92 units	<20 units
Complement component 3 (C3)	1.213 g/L	0.811–1.570 g/L
Complement component 4 (C4)	0.37 g/L	0.129–0.392 g/L
Cryoglobulins	Absent	Absent
Thyroid-stimulating hormone (TSH)	0.84 mIU/L	0.35–4.94 mIU/L
Immunoglobulin G (IgG)	12.40 g/L	6.103–16.6 g/L
Immunoglobulin A (IgA)	2.32 g/L	0.84–4.99 g/L
Immunoglobulin M (IgM)	1.47 g/L	0.35–2.42 g/L

Allergy and immunology assessment, prompted by an acute reaction comprising angioedema, dizziness, palpitations, chest pain, and dyspnea within minutes of ingesting legumes and nuts, confirmed IgE-mediated food allergy. He additionally reported post-nasal drip, purulent discharge, facial fullness, and headache, consistent with chronic rhinosinusitis without nasal polyps.

Cross-sectional imaging at presentation on neck ultrasound demonstrated an enlarged right submental lymph node measuring 1.9 × 0.9 cm, with baseline computed tomography (CT) advised. Neck CT showed a large, inhomogeneously enhancing soft-tissue mass in the right submandibular region, separable from and displacing the submandibular gland medially, measuring approximately 4.2 × 2.3 cm, together with multiple enhancing lymph nodes at levels IIa/b and III on the right, the largest measuring 1.3 cm in short axis, findings reported as highly suggestive of a lymphoproliferative process (Figure 2). Staging chest CT showed bilateral nodular and ground-glass opacities felt to reflect an underlying infectious or inflammatory process, without suspicious nodules, masses, or intrathoracic lymphadenopathy. Abdominal and pelvic CT identified indeterminate hepatic hypodensities; dedicated follow-up magnetic resonance imaging one month later showed a non-cirrhotic liver with multifocal subcapsular bilobar lesions, fewer in number than on the prior CT, considered most consistent with a resolving infectious or inflammatory process, including possible diffuse eosinophilic infiltration, given the known peripheral eosinophilia, or granulomatous hepatitis.

**Figure 2:**
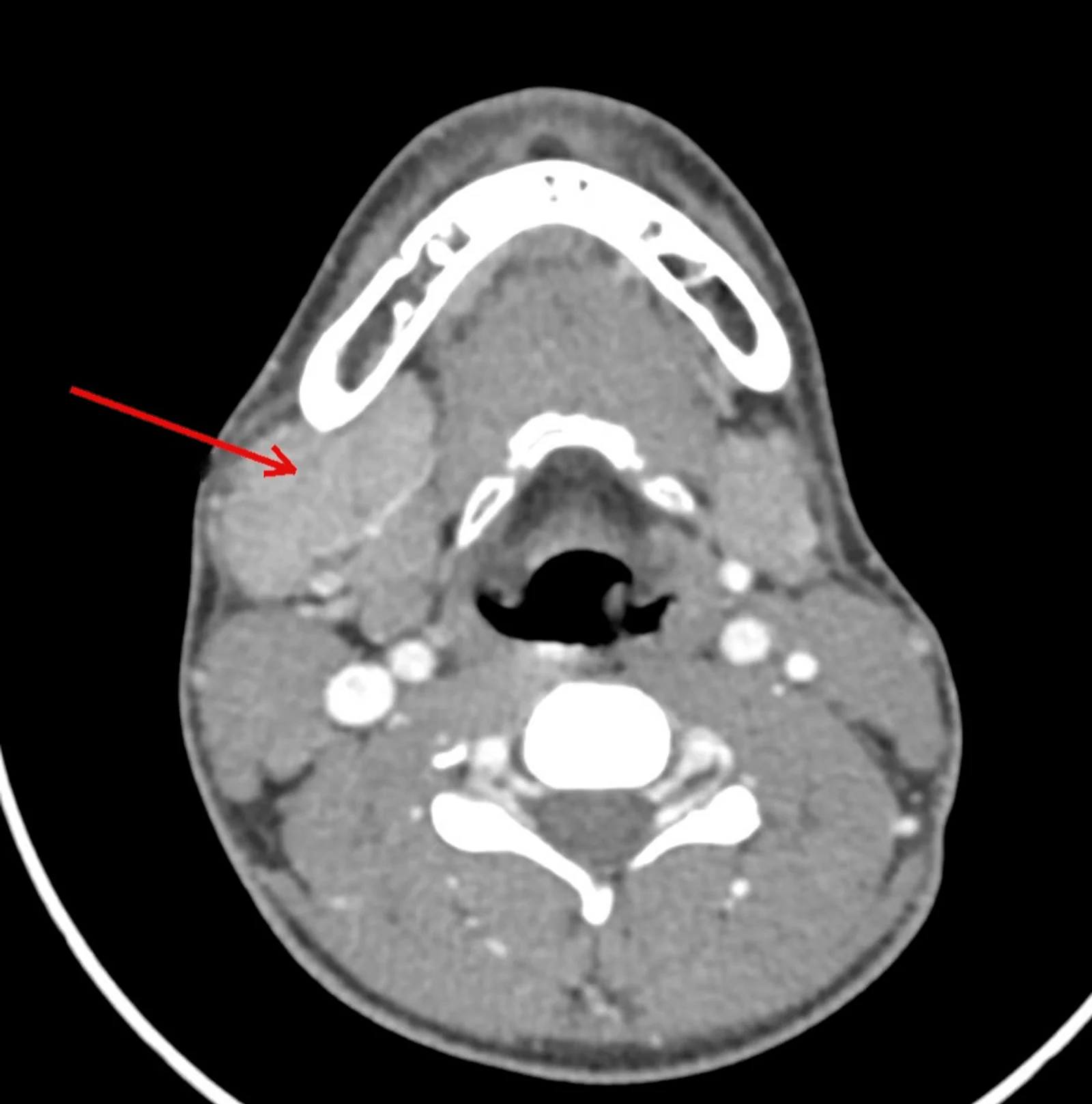
Axial contrast-enhanced computed tomography of the neck at presentation. The red arrow indicates the large, inhomogeneously enhancing right submandibular soft-tissue mass.

Pathological evaluation

First Core-Needle Biopsy

Ultrasound-guided core biopsy of the right submandibular tissue showed reactive lymphoid hyperplasia with focal microabscess and granuloma formation. Special stains, including periodic acid-Schiff (PAS), Grocott methenamine silver (GMS), and acid-fast, were negative for micro-organisms. Immunohistochemistry demonstrated preserved reactive architecture - CD20/CD10/BCL6-positive, BCL2-negative germinal-center B cells surrounded by CD3/CD5-positive T cells, intact CD21/CD23 follicular dendritic meshworks, negative CD30, and a reactive Ki-67 proliferation pattern - without cytologic atypia or necrosis. Excisional biopsy was recommended should clinical suspicion persist.

Second Core-Needle Biopsy

Given persistent lymphadenopathy, a repeat image-guided core biopsy (five passes) was performed approximately two months later. The submitted tissue was small and composed predominantly of small lymphocytes with collections of eosinophils and prominent small vessels. The sample again proved insufficient for a definitive diagnosis, and excisional biopsy was strongly advised.

Excisional Lymph Node Biopsy

A right submandibular lymph node measuring 4.0 × 3.5 × 1.5 cm was excised. Histology showed enlarged, well-spaced secondary lymphoid follicles maintaining the normal zoning pattern of follicle center cells with an intact mantle zone; a paracortex expanded by numerous eosinophils accompanied by small lymphocytes and occasional immunoblasts; eosinophilic microabscesses, with eosinophilic proteinaceous material also infiltrating some follicle centers; paracortical vascular proliferation; and a focus of Charcot-Leyden crystals. The perinodal connective tissue contained scattered lymphoid follicles and sheets of eosinophils. No malignant cells of any type were identified. A broad immunohistochemical panel (CD20, PAX-5, CD3, CD5, CD4, CD8, CD30, CD15, BCL2, S100, CD68, Ki-67) confirmed a polymorphous, reactive pattern without evidence of lymphoma, and staining for Epstein-Barr virus (EBV)-encoded small RNA (EBER) and human herpesvirus 8 (HHV-8) was negative. The findings were independently reviewed and confirmed by two consultant pathologists. Together with the peripheral eosinophilia, these features were diagnostic of KD (Figure 3).

**Figure 3:**
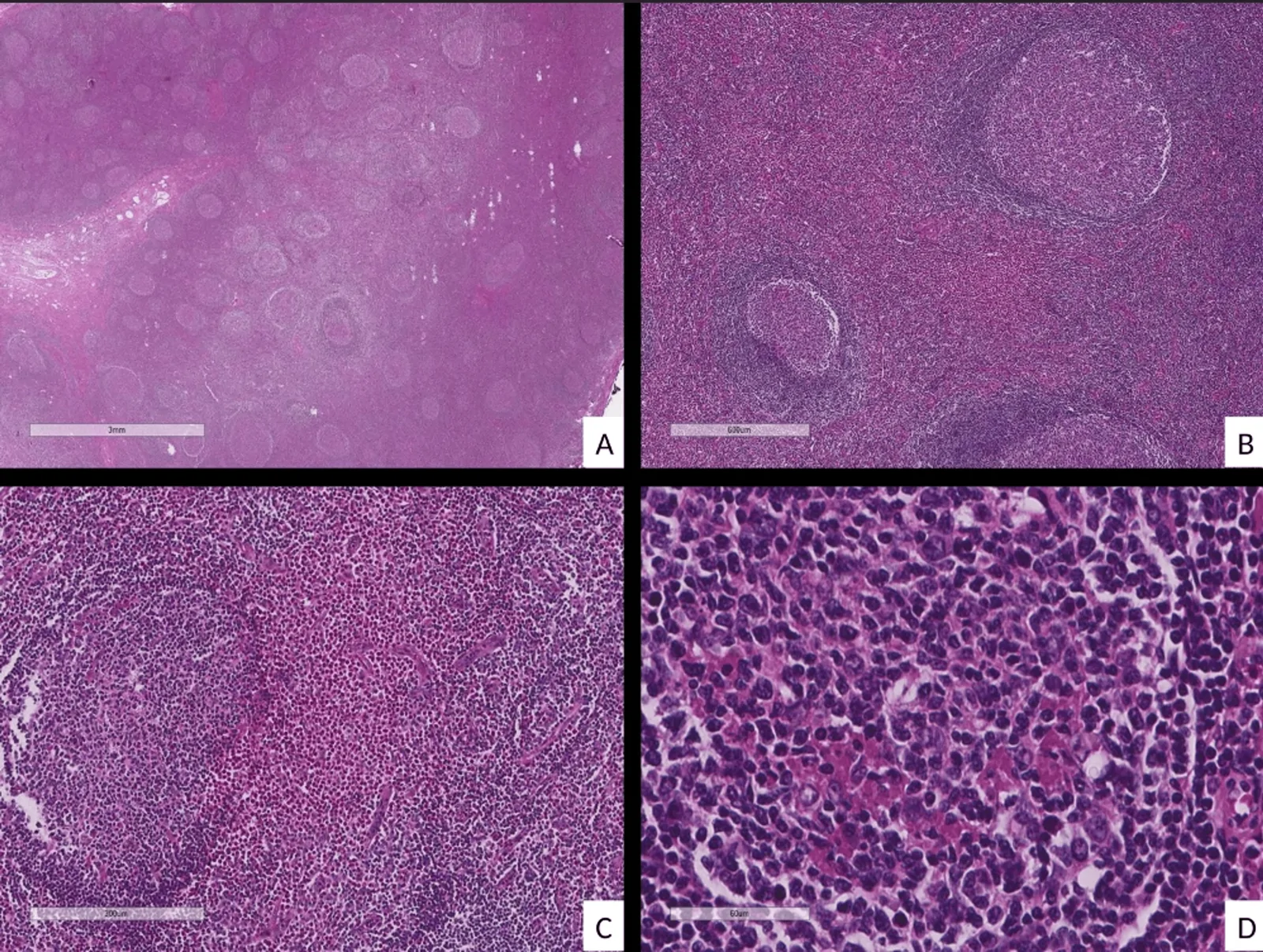
Histopathology of the excised right submandibular lymph node. (A) Low-power view showing intact secondary lymphoid follicles with preserved germinal centers (H&E, low power; scale bar 3 mm). (B) Enlarged, well-spaced follicles with an expanded paracortex (H&E, scale bar 600 µm). (C) Paracortical eosinophilic microabscess with follicular eosinophilic infiltration (H&E, scale bar 300 µm). (D) High-power view of eosinophil-rich infiltrate with eosinophilic proteinaceous material (H&E, scale bar 60 µm).

Diagnosis

Kimura disease, established by excisional lymph node biopsy following two non-diagnostic core-needle biopsies, in an adolescent with pre-existing T1DM, IgE-mediated food allergy, and chronic rhinosinusitis.

Therapeutic intervention and follow-up

Excision of the involved submandibular lymph node was both diagnostic and locally therapeutic. Systemic corticosteroids were used subsequently for disease control. Food allergen avoidance with a written allergy action plan and intranasal corticosteroid therapy were instituted for the concurrent atopic manifestations, and diabetes management continued unchanged under pediatric endocrinology with insulin-pump therapy and reinforced diabetes education.

The patient remains under longitudinal multidisciplinary follow-up encompassing rheumatology, hematology, allergy and immunology, otolaryngology, and endocrinology. Given that recurrence rates in KD after surgical excision are substantial, ongoing clinical and laboratory surveillance, including serial eosinophil counts, serum IgE, and urinalysis for proteinuria to detect renal involvement, has been maintained.

## Discussion

This case documents a clinically and histologically classic presentation of KD in an adolescent with established T1DM. Every cardinal feature of KD was present: a young male patient, a painless and slowly progressive unilateral submandibular mass with regional cervical lymphadenopathy, marked peripheral eosinophilia, serum IgE persistently above 5000 IU/mL, and diagnostic nodal histology comprising preserved follicular architecture, paracortical eosinophilic infiltration with microabscess formation, Charcot-Leyden crystals, and proliferation of postcapillary venules [1-3]. What distinguishes this report is the immunologic context in which that classic disease arose.

KD is conventionally regarded as a Th2-driven allergic or hypersensitivity disorder rather than an autoimmune one, and its principal recognized systemic association is renal - nephrotic syndrome occurring in approximately 10-20% of patients, with mesangioproliferative glomerulonephritis, membranous nephropathy, and minimal-change disease all described [4,5]. The observation that renal disease accompanies KD in a meaningful minority of patients has prompted the suggestion that KD may represent one manifestation of a wider systemic immunologic disturbance rather than a purely localized allergic phenomenon [1,5]. Our patient extends this observation: his KD arose against a background of established organ-specific autoimmunity in the form of T1DM, a striking familial autoimmune burden encompassing type 1 diabetes in a sibling and two maternal cousins, multiple sclerosis in a maternal cousin, and thyroid disease in a maternal uncle, together with IgE-mediated food allergy and chronic rhinosinusitis.

Historically, the coexistence of a Th1-mediated disease such as type 1 diabetes and a Th2-driven disorder such as KD would have appeared paradoxical under the classical Th1/Th2 counter-regulatory paradigm, in which polarization toward one axis was thought to suppress the other. That model has been substantially revised. Contemporary understanding recognizes additional effector lineages and, critically, that impaired regulatory T-cell function can permit both Th1- and Th2-driven pathology to emerge in the same host [7]. From this perspective, the concurrence observed here is better interpreted not as a coincidence of two unrelated rare events, but as the expression of a generalized failure of immune tolerance in a genetically predisposed individual - the same framework that explains the well-established clustering of type 1 diabetes with autoimmune thyroid and celiac disease. Whether this association is causal or coincidental cannot be determined from a single observation. KD is rare, and chance co-occurrence with a common childhood autoimmune disease is entirely possible. We report it because the combination appears not to have been previously described, and because recognition of such associations is the necessary first step toward determining whether they are more than coincidental.

From a practical standpoint, this case also reinforces the well-recognized limitation of needle sampling in KD. Our patient underwent two separate ultrasound-guided core-needle biopsies over several months, yielding only non-specific reactive changes, before excisional biopsy secured the diagnosis. This reflects the fact that the diagnosis depends on architectural features appreciable only across an intact node, preserved follicular zonation, paracortical eosinophilic microabscesses, and vascular proliferation, which cannot be reliably assessed in fragmented cores [2,3,8]. In a young patient with a slowly enlarging neck mass and peripheral eosinophilia, and particularly where imaging raises the possibility of lymphoproliferative disease, we would advocate proceeding to excisional biopsy early rather than after successive inconclusive needle passes. The presence of diabetes adds a further consideration, since both the disease and any subsequent corticosteroid therapy carry implications for glycemic control that warrant coordinated endocrinology input.

Management of KD comprises surgical excision, corticosteroids, or both, with radiotherapy and immunosuppressive agents reserved for refractory disease; recurrence after excision alone is reported in up to 50-60% of cases, mandating sustained follow-up [1]. For genuinely refractory or relapsing disease, an expanding body of case-level evidence supports biologic agents targeting type 2 inflammation, including anti-IL-5 and anti-IL-4 receptor alpha therapies [9,10]. In a patient with concurrent type 1 diabetes, such steroid-sparing options are of particular interest given the metabolic penalty of repeated or prolonged corticosteroid exposure.

The principal limitation of this report is inherent to its single-patient design: an association observed once cannot establish a pathogenic link, and the immunologic interpretation offered here is necessarily inferential. Longer follow-up will be required to characterize the disease evolution in this patient.

## Conclusions

We describe a classic presentation of KD in an adolescent with established T1DM, a co-occurrence that, to our knowledge, has not previously been reported. The case supports the view that KD may arise within a broader landscape of immune dysregulation, here encompassing organ-specific autoimmunity, a marked familial autoimmune burden, and coexisting atopy, rather than as an isolated allergic phenomenon. Clinically, it reinforces that KD belongs in the differential diagnosis of persistent cervical lymphadenopathy with eosinophilia in adolescents, and that excisional biopsy should be pursued early, since core-needle sampling is frequently non-diagnostic and may substantially delay recognition.
